# Selective but not pan-CDK inhibition abrogates 5-FU-driven tissue factor upregulation in colon cancer

**DOI:** 10.1038/s41598-024-61076-5

**Published:** 2024-05-08

**Authors:** Annika Kayser, Annabell Wolff, Peggy Berlin, Lara Duehring, Larissa Henze, Ralf Mundkowski, Wendy Bergmann, Brigitte Müller-Hilke, Charlotte Wagner, Maja Huehns, Sonja Oehmcke-Hecht, Claudia Maletzki

**Affiliations:** 1 Department of Medicine, Clinic III - Hematology, Oncology, Palliative Medicine, Rostock University Medical Center, Ernst-Heydemann-Str. 6, 18057 Rostock, Germany; 2 Department of Medicine II, Division of Gastroenterology, Rostock University Medical Center, 18057 Rostock, Germany; 3 Institute of Medical Microbiology, Virology and Hygiene, Rostock University Medical Center, 18057 Rostock, Germany; 4 Department of Internal Medicine II, Asklepios Hospital Harz, Goslar, Germany; 5 Center of Pharmacology and Toxicology, Institute of Clinical Pharmacology, Rostock University Medical Center, 18057 Rostock, Germany; 6 Laboratory for Clinical Immunology, Core Facility for Cell Sorting and Cell Analysis, Rostock University Medical Center, 18057 Rostock, Germany; 7 Institute of Pathology, Rostock University Medical Center, 18057 Rostock, Germany

**Keywords:** Epithelial-mesenchymal transition, Senescence, Targeted therapy, Cell sorting, Hypercoagulation, Microvesicles, Cancer, Medical research, Oncology

## Abstract

Thromboembolic events are complications in cancer patients and hypercoagulability has been linked to the tissue factor (TF) pathway, making this an attractive target. Here, we investigated the effects of chemotherapeutics and CDK inhibitors (CDKI) abemaciclib/palbociclib (CDK4/6), THZ-1 (CDK7/12/13), and dinaciclib (CDK1/2/5/9) alone and in combination regimens on TF abundance and coagulation. The human colorectal cancer (CRC) cell line HROC173 was treated with 5-FU or gemcitabine to stimulate TF expression. TF^+^ cells were sorted, recultured, and re-analyzed. The effect of treatment alone or in combination was assessed by functional assays. Low-dose chemotherapy induced a hypercoagulable state and significantly upregulated TF, even after reculture without treatment. Cells exhibited characteristics of epithelial-mesenchymal transition, including high expression of vimentin and mucin. Dinaciclib and THZ-1 also upregulated TF, while abemaciclib and palbociclib downregulated it. Similar results were observed in coagulation assays. The same anticoagulant activity of abemaciclib was seen after incubation with peripheral immune cells from healthy donors and CRC patients. Abemaciclib reversed 5-FU-induced TF upregulation and prolonged clotting times in second-line treatment. Effects were independent of cytotoxicity, senescence, and p27^kip1^ induction. TF-antibody blocking experiments confirmed the importance of TF in plasma coagulation, with Factor XII playing a minor role. Short-term abemaciclib counteracts 5-FU-induced hypercoagulation and eventually even prevents thromboembolic events.

## Introduction

Thromboembolic complications occur in approximately 50% of cancer patients (i.e. *Trousseau syndrome*)^1–3^. This life-threatening, yet preventable side effect is very complex and results from tumor-derived vesicles and growth factors, vascular wall leakage, and impaired blood flow, among other factors^4,5^. In colorectal cancer (CRC), cancer-related thrombosis risk is moderate. However, it increases with surgery and chemotherapy because the cancer cells interact with the coagulation system, platelets, and the vascular endothelium^6^. Platelets are activated by CRC cells via endogenous and exogenous factors^7^. These, in turn, release extracellular vesicles that are involved in the activation of hemostasis and ultimately contribute to the prothrombotic phenotype of CRC patients^8,9^.

Intravascular tissue factor (TF) initiates blood coagulation^10^. TF is found on subendothelial cells, such as smooth muscle cells and fibroblasts, but it is also highly inducible on monocytes, endothelial cells, and the cancer vasculature. In the latter, TF regulates intracellular signaling pathways and stimulates cancer cell growth, invasion, and dissemination^11,12^. The central role of TF in the coagulation system reflects its relevance in cancer coagulopathy. Several studies have shown that the cancer cell itself is a source of TF^13^. Molecularly, TF expression results from mutational events in tumor suppressors and oncogenes, epithelial-mesenchymal transition (EMT), and hypoxia. In CRC, *K-ras* and *TP53* mutations and epidermal growth factor receptor activation are primarily responsible for TF up-regulation via MAPK and PI3K signaling pathways. TF is also involved in interactions with cytoskeletal proteins and in protecting circulating cancer cells from NK cell lysis^14^. TF abundance correlates with disease progression^15^, allowing the formation of blood-conducting tubes not lined by endothelial cells, so-called vasculogenic mimicry^16^. As such, TF expression is a marker of poor prognosis or CRC recurrence^17^. Tumor cell-bound and circulating TF can be detected in cancer patients^8,10^. In the latter, TF is usually released from tumor cells via microvesicles (MVs)^18,19^. MVs can be used as prognostic biomarkers^18,20,21^. However, few studies have evaluated the association between MV-TF activity and venous thromboembolism in cancer patients. In general, there is a wide variation between patients and also between different tumor types. In CRC patients, most studies do not show a direct association between MV-TF activity and venous thromboembolism. While TF is required for the malignant phenotype of CRC, it is unknown whether and to what extent chemotherapy itself induces TF on cancer cells and what the biological consequences of TF upregulation are. Here, we identified a massive upregulation of TF upon low-dose chemotherapy, which was stable after TF^+^ cell sorting and drug-free reculture. Notably, TF^+^ and intrinsically “drug resistant” cells exhibited accelerated clotting time and had an EMT-like phenotype. Using different combination approaches with targeted agents, we were able to show that selective, but not pan-CDK inhibitors abolished the procoagulant behavior via TF downregulation. This study highlights the relevance of TF in malignancy and further supports its involvement in cancer progression, in addition to its relevance in non-hemostatic functions.

## Results

### Chemotherapy upregulates TF on low-passage CRC cells

**Figure 1:**
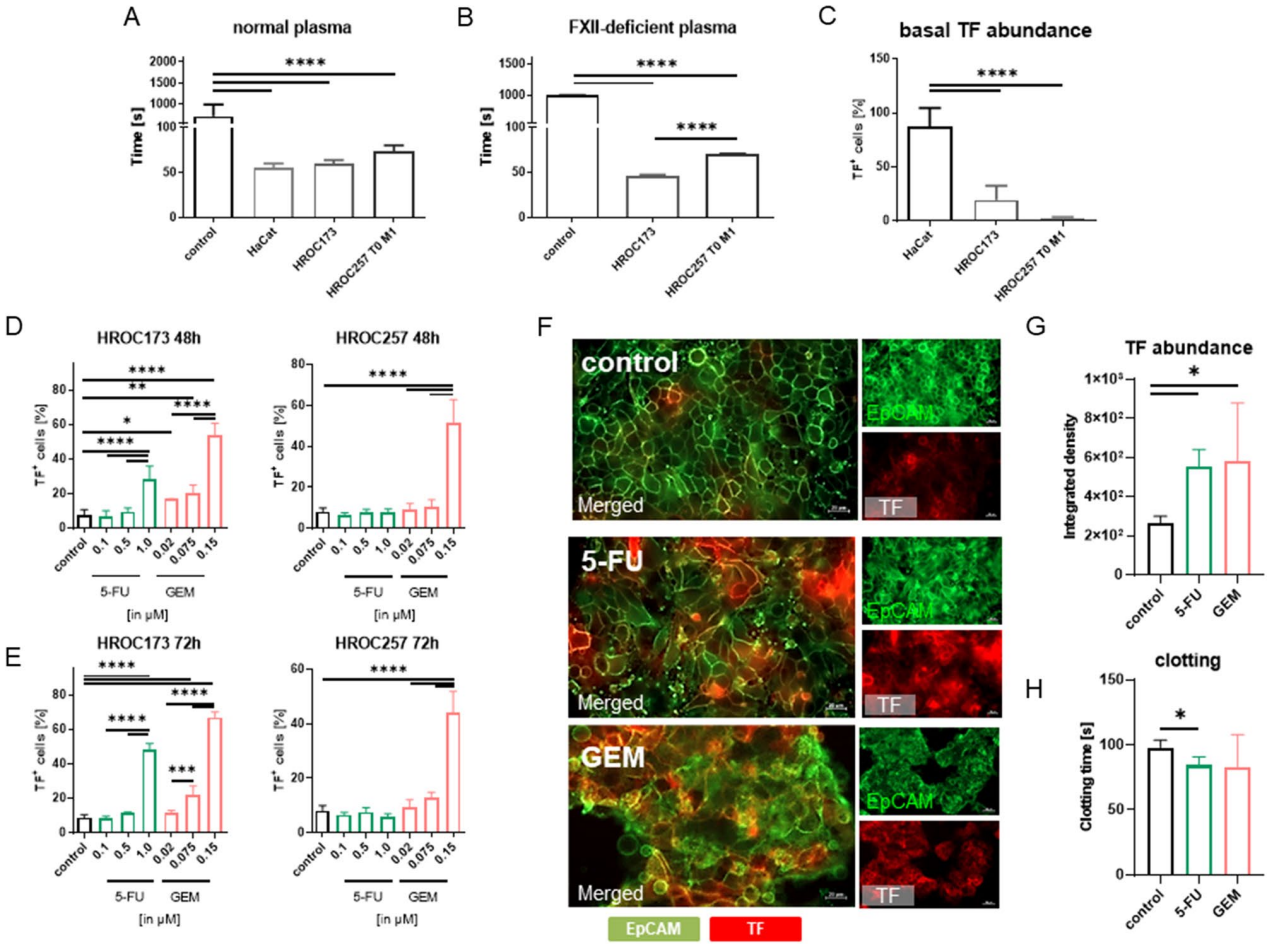
Plasma coagulation time and TF abundance on tumor cells. (**A**) normal plasma and (**B**) FXII-deficient plasma, n ≥ 3; mean + SD; one-way ANOVA (Tukey's multiple comparison test). ****p < 0.0001 versus control. (**C**) Basal and (**D**,**E**) drug-induced TF abundance on different tumor cell lines. Tumor cells were left untreated or exposed to increasing doses of 5-FU (0.1, 0.5, and 1.0 µM, respectively) or gemcitabine (GEM, 0.02, 0.075, and 0.15 µM, respectively) for 48 or 72 h, n = 3–4; mean + SD; one-way ANOVA (Tukey's multiple comparison test). ****p < 0.0001; **p < 0.01; *p < 0.05 versus control. (**F**) Immunofluorescence. Tumor cells were either left untreated or exposed to 1 µM 5-FU and 0.15 µM GEM for 48 h. Green—EpCAM; red—TF. (**F**) Representative images. Original magnification ×200. Scale bar: 20 µm. (**G**) Quantification of TF intensity, n = 3; mean + SD; one-way ANOVA (Tukey's multiple comparison test). *p < 0.05 versus control. (H) Influence of selected drugs (1.0 µM 5-FU, 0.15 µM GEM) on plasma coagulation time, n ≥ 3; mean + SD; one-way ANOVA (Tukey's multiple comparison test). *p < 0.05 versus control.

Plasma coagulation in normal and FXII-deficient plasma, spontaneous release of TF, and the amount of membrane-bound TF were investigated (Fig. 1). We used two different CRC cell lines HROC173 and HROC257 T0 M1, HaCat cells served as a positive control (Fig. 1A–D). CRC cell lines were established from sporadic CRC with chromosomal instability (= HROC173, *KRAS*^*mut*^*, TP53*^*mut*^) or microsatellite instability (= HROC257 T0 M1, *KRAS*^*wt*^*, TP53*^*mut*^), covering the two main CRC molecular subtypes.

Coagulation times in normal plasma were comparable between cells, with cell line-specific differences (Fig. 1A). Equal clotting times were determined in FXII-deficient plasma with the CRC cells HROC173 and HROC257 T0 M1 (Fig. 1B), suggesting a minor role of the intrinsic coagulation cascade under basal conditions. However, basal TF abundance on CRC cells was significantly lower, compared to HaCat cells (HROC173 > HROC257 T0 M1; Fig. 1C).

The effect of the clinical drugs 5-FU and Gemcitabin (GEM) was then investigated. Both drugs significantly upregulated TF on HROC173 and HROC257 T0 M1 cells in a dose-dependent manner (Fig. 1D,E). However, there was no time-dependent effect as TF upregulation was already detectable after 48h and remained high after 72h exposure to 5-FU (1.0 µM) or GEM (0.15 µM) (*p* < 0.0001 vs. control and low-dose, respectively, Fig. 1D,E). To test whether the chemotherapy-induced upregulation of TF was associated with direct toxicity or impaired migration, crystal violet staining was done, accompanied by a classical wound healing assay with the highest dose of each compound, i.e. 1 µM 5-FU and 0.15 µM GEM, respectively (Supplementary Fig. 1). No cell death or impaired migration was observed, excluding direct cytotoxicity of the drug doses used. A closer look at the two CRC lines revealed cell line-specific effects. HROC173 cells responded with greater TF upregulation, after both 5-FU and GEM treatment (Fig. 1F,G), compared to HROC257 T0 M1, in which TF was higher only after GEM exposure. Clotting times were significantly shorter when 5-FU-treated HROC173 cells were used for coagulation of plasma (Fig. 1H). Since most CRC cases show chromosomal instability, all subsequent experiments were performed with HROC173.

### Stable TF upregulation after single chemotherapy of CRC cells

**Figure 2:**
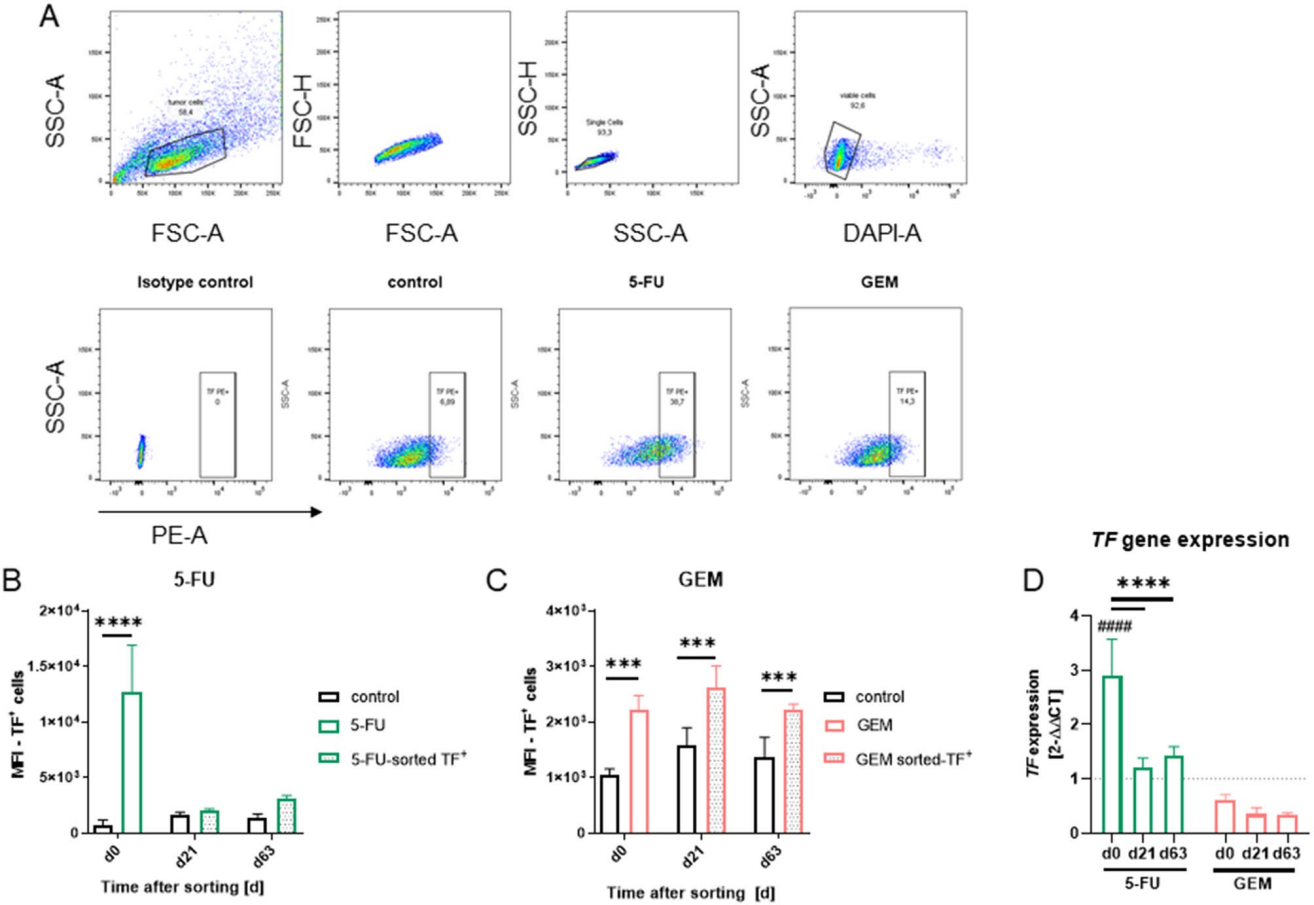
Cell sorting approach, culture of TF^+^ cells and re-analysis. CRC cells were treated with 5-FU (1 µM) or GEM (0.15 µM) for 48h. Control cells were left untreated. Afterwards, the cells were harvested and stained with an PE anti-CD142 antibody. Dead exclusion was done with DAPI. Based on the mean fluorescence intensity (MFI), the top 20% CD142-positive cells (= TF^high^-positive cell fraction) were bulk sorted on a BD FACSAria™IIIu. (**A**) Representative dot blots of TF^+^ cells for each condition. (**B**,**C**) Quantitative analysis of TF-abundance at day 21 and day 63 after sorting compared to untreated cells which were cultured in parallel to the TF^+^ cell fraction. The mean fluorescence intensity (MFI) is given. All experiments were run in triplicates and repeated at least three times; mean + SD; two-way ANOVA (Sidak's multiple comparison test). ****p < 0.0001; ***p < 0.001 versus control. (**D**) TF gene expression analysis, n ≥ 3 independent experiments. Mean + SD; two-way ANOVA (Sidak's multiple comparison test). ^####^*p* < 0.0001 versus untreated cells; ****p < 0.0001 versus d0.

We then investigated whether the drug-induced TF-upregulation was the sole result of an acute cellular stress response or whether it was stable after drug withdrawal. A sorting approach was initiated and TF^+^ cells (= initially drug resistant) with the highest TF expression (MFI ≥ 20%, approximately 5%) were collected (Fig. 2A). TF^+^ cells were recultured without drugs and analyzed twice on days 21 and 63 (Fig. 2B–D). Analysis confirmed massive TF upregulation after single, low-dose chemotherapy. MFI increased significantly after 5-FU or GEM treatment, but decreased after reculture (d21, d63, Fig. 2B,C). Likewise, TF gene expression levels were initially 2.5-fold higher after 5-FU treatment than in controls (Fig. 2D). GEM had no effect on TF expression (Fig. 2D).

### Short-term chemotherapy induces genes involved in epithelial-mesenchymal transition

**Figure 3:**
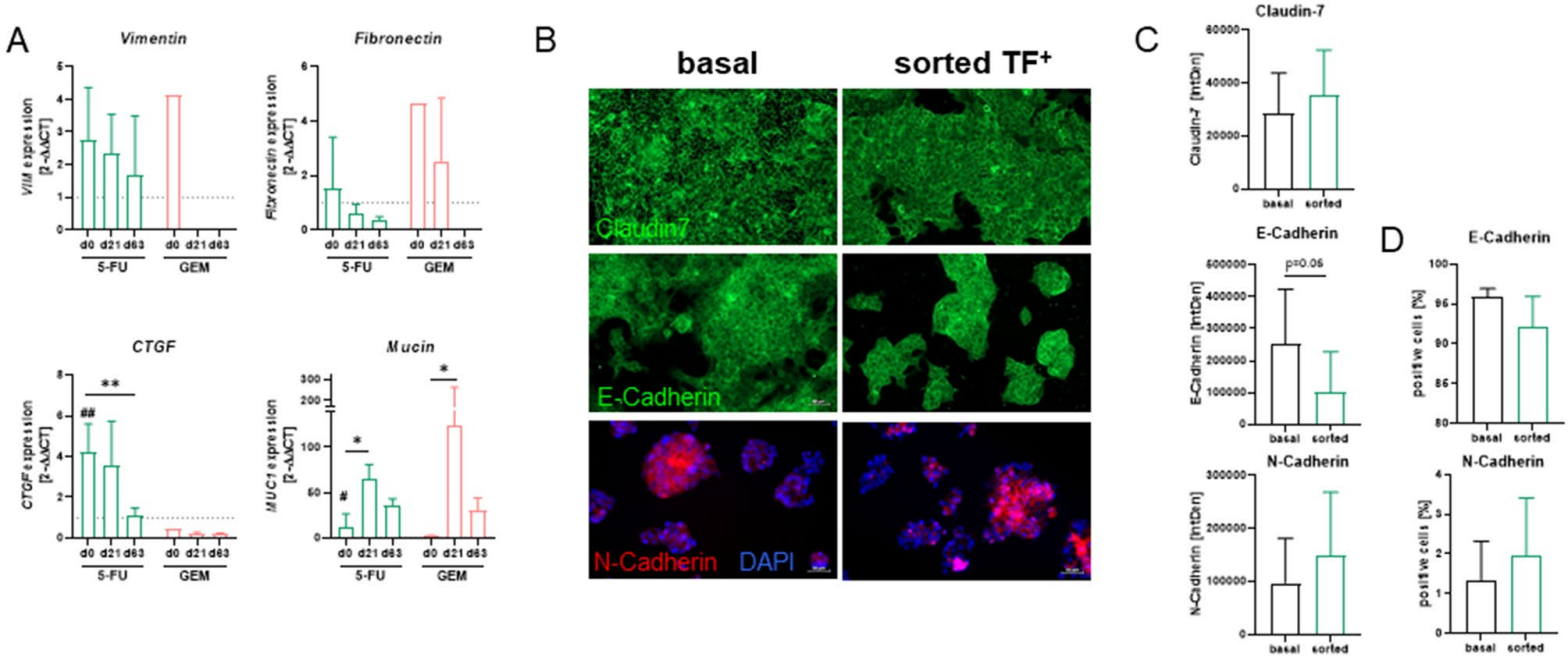
EMT marker using gene expression analysis, immunofluorescence, and flow cytometry. (**A**) Gene expression analysis (2^−ΔΔCT^), n ≥ 3; mean + SD; two-way ANOVA (Sidak's multiple comparison test). ^##^*p* < 0.01 versus untreated cells (d0 indicates after 48h treatment, but without reculture); **p < 0.01; *p < 0.05 versus control. (**B**,**C**) Immunofluorescence was done to study abundance of Claudin-7 and E-Cadherin, as well as N-Cadherin. (**B**) Representative images of tumor cells to illustrate basal protein abundance and 5-FU-induced changes on sorted TF^+^ cells. (**C**) Quantification, n ≥ 3; mean + SD. (**D**) Quantification of E-Cadherin and N-Cadherin on HROC173 cells was additionally done by flow cytometry to confirm immunofluorescence data, n = 3; mean + SD.

TF upregulation can increase tumor growth and the metastatic potential of tumor cells. EMT is the first step, and is characterized by gene expression and morphological changes. Short-term treatment with 5-FU and GEM resulted in upregulation of *Vimentin*, *Fibronectin*, *CTGF,* and *Mucin-1* (Fig. 3A). Notably, expression levels remained higher in TF^+^ sorted cells than in controls even after reculture without treatment (Fig. 3A). The effects were more pronounced in 5-FU-treated cells than in GEM-treated cells. EMT characteristics were partially confirmed in immunofluorescence (Fig. 3B,C). However, the differences did not reach statistical significance due to the standard deviation (Fig. 3C). Additional flow cytometry confirmed slightly altered E-Cadherin/N-Cadherin ratios, indicative of EMT (Fig. 3D). Again, no significant changes in the abundance of E-Cadherin and N-Cadherin were observed.

### CDK inhibitors have different effects on hypercoagulability

**Figure 4:**
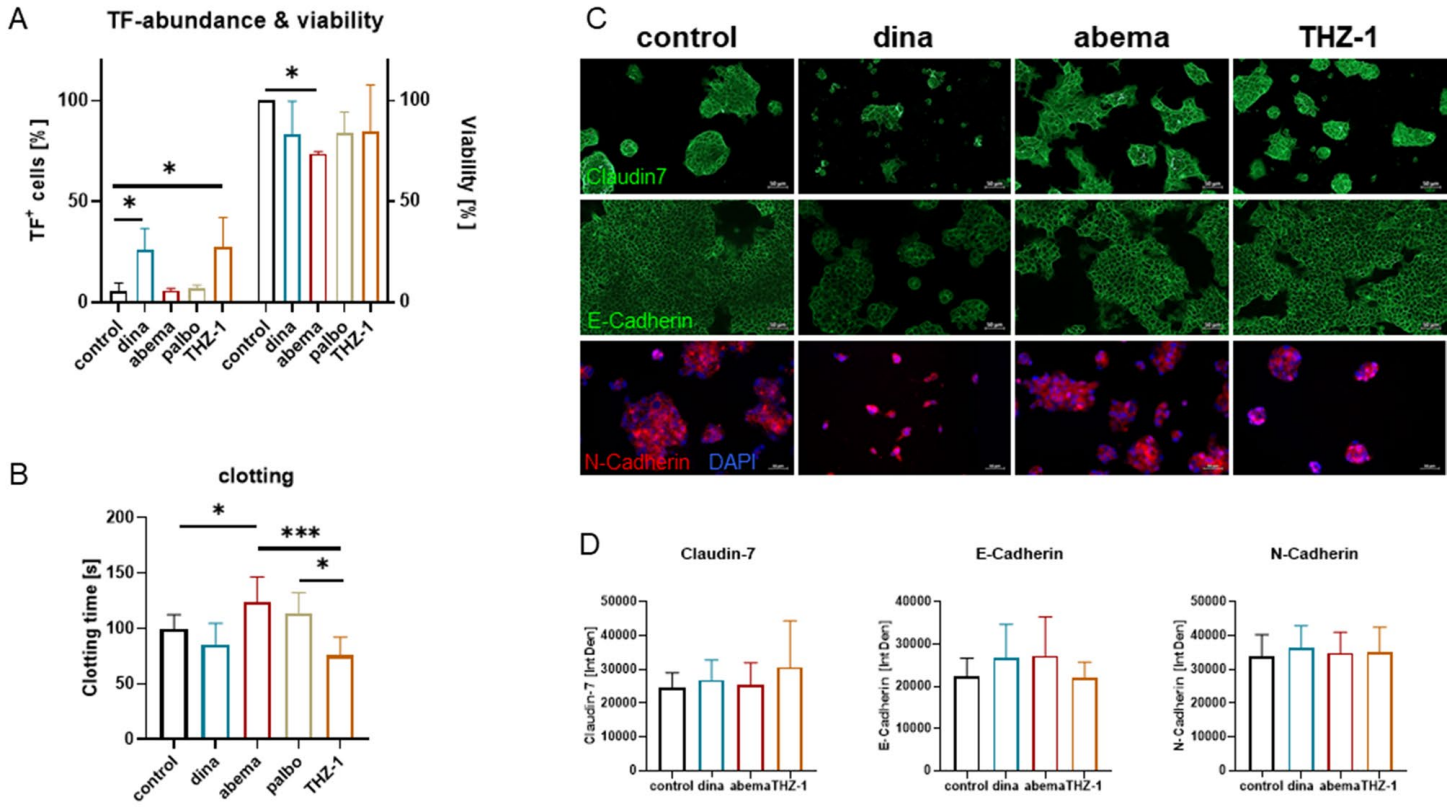
TF abundance, coagulation time, and EMT after CDKI treatment. Tumor cells were either left untreated or exposed to CDKIs (dinaciclib 0.01 µM, abemaciclib 1 µM, palbociclib 1 µM, and THZ-1 0.03 µM) for 48 h. (**A**) Flow cytometric TF quantification and assessment of viability (crystal violet staining) after CDKI treatment in comparison to untreated control cells, n ≥ 3; one-way ANOVA (Tukey's). *p < 0.05 versus control. (**B**) Coagulation time, n ≥ 5; mean + SD; one-way ANOVA (Sidak's multiple comparison test). ***p < 0.001; **p < 0.01; *p < 0.05 versus. control. (**C**,**D**) Claudin-7, E-Cadherin, and N-Cadherin immunofluorescence. (**C**) Representative images of tumor cells to illustrate basal and treatment-induced expression. No significant changes in any of the markers were observed. (D) Quantification, n ≥ 3; mean + SD.

Above results confirmed the increased procoagulant properties of classical chemotherapy. Hence, we tested whether this effect is also present after targeted therapy with CDK inhibitors (CDKI) (Fig. 4A,B). All drugs were used at doses below IC_50_ to exclude the possibility that toxic effects are the only explanation for any changes (Fig. 4A). With the four CDKIs included here, dinaciclib, palbociclib, and THZ-1 had no effect on viability, while abemaciclib reduced cell viability to ~ 75%.

The four CDKI had individual effects on TF abundance (Fig. 4A). Pan-CDKI Dinaciclib and CDK7I THZ-1 significantly upregulated TF (*p* < 0.05 vs. control) to a degree comparable to chemotherapeutics. By contrast, CDK4/6Is abemaciclib and palbociclib did not change TF expression on residual cells (Fig. 4A). Moreover, the plasma coagulation time (Fig. 4B) was significantly prolonged after abemaciclib, compared to non-treated cells (Fig. 4A). Palbociclib also had a trend toward prolongation of clotting time. Conversely, dinaciclib, and THZ-1 increased the procoagulant activity as evidenced by a reduction in plasma coagulation time. No effect on EMT was observed (Fig. 4C,D).

Cellular hyper- or hypocoagulability may either result from altered p27^Kip1^ expression or senescence. The former has previously been reported to regulate TF expression and activity in endothelial cells^22^ and the latter has been directly linked to the coagulation pathway^23,24^. Protein levels of the cell cycle inhibitor p27^Kip1^ were altered after treatment with dinaciclib and abemaciclib (Supplementary Fig. 2A, D). 5-FU and THZ-1 had no effect on p27^Kip1^ abundance (Supplementary Fig. 2A, D). Senescence was not induced by either treatment (Supplementary Fig. 2B–D). Therefore, no correlation between coagulation and senescence was observed in this study.

### Sequential, but not simultaneous combination therapy abrogates the procoagulant activity of CRC cells

**Figure 5:**
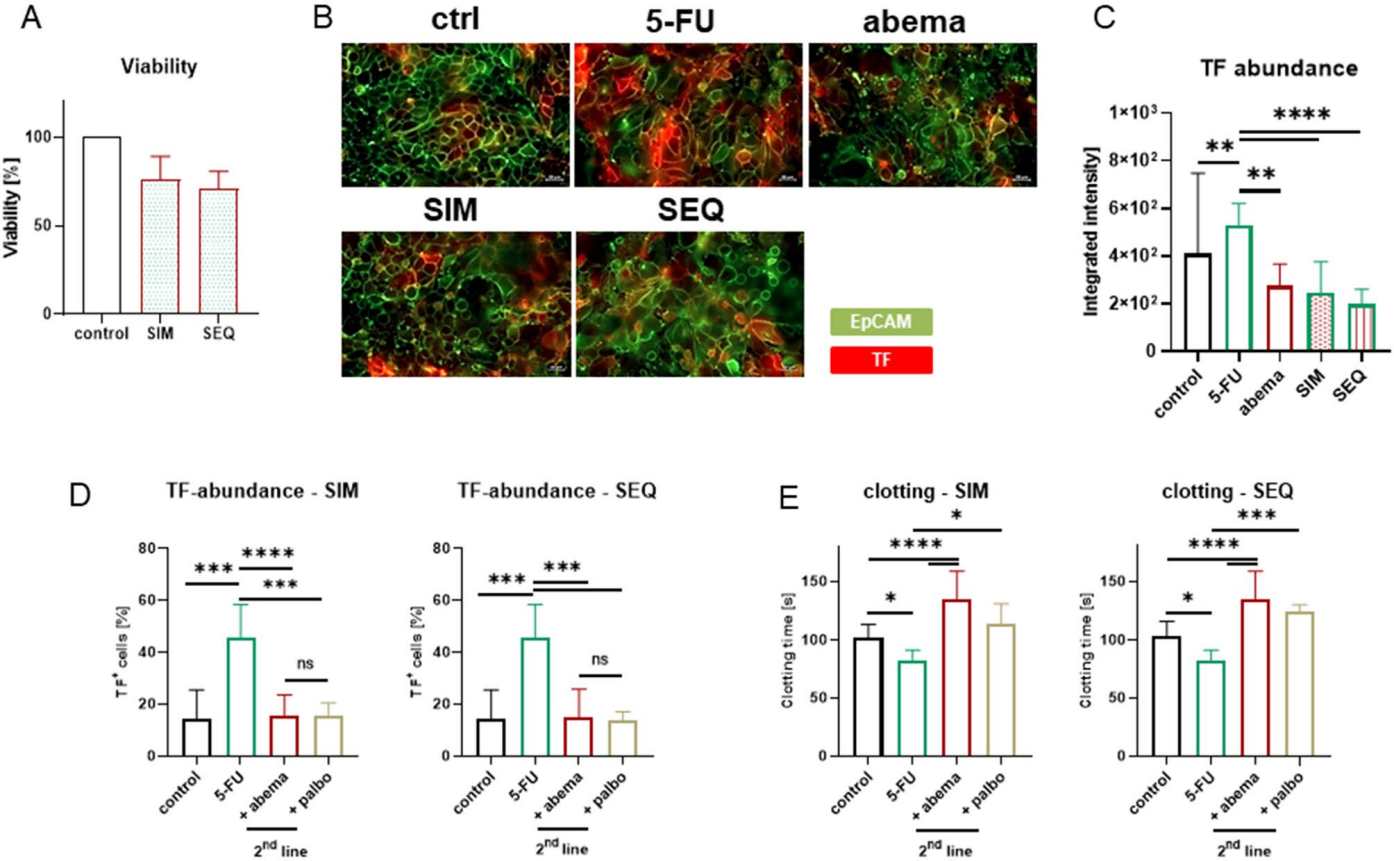
TF abundance and coagulation time after combination treatment. Two different approaches were studied: simultaneous (SIM) and sequential (SEQ) treatment. For the latter, tumor cells were exposed to 5-FU (1 µM), followed by abemaciclib (1 µM). In some experiments, palbociclib was included to compare effects between two different CDK4/6Is. (**A**) Assessment of viability (crystal violet staining) after combined 5-FU/abema treatment in comparison to untreated control cells to exclude enhanced toxicity in the combination, n ≥ 3, mean + SD. (**B**,**C**) Immunofluorescence. Green—EpCAM; red—TF. (**B**) Representative images are shown. Original magnification ×200. Scale bar: 20 µm. (**C**) Quantification, n = 3; mean + SD; one-way ANOVA (Tukey's multiple comparison test). ****p < 0.0001; **p < 0.01 versu control. (**D**) Flow cytometric TF quantification, n ≥ 5; mean + SD; one-way ANOVA (Tukey's multiple comparisons test). ****p < 0.0001; ***p < 0.001 versus control. (**E**) Coagulation time, n ≥ 5; mean + SD; one-way ANOVA (Tukey's multiple comparisons test). ****p < 0.0001; ***p < 0.001; *p < 0.05 versus control.

Combination strategies were used to investigate the beneficial effect of abemaciclib on the procoagulant activity of CRC cells. Simultaneous (SIM) or sequential (SEQ) treatments were used to test whether 5-FU induced TF up-regulation could be suppressed by SIM treatment or even reversed by SEQ abemaciclib treatment (Fig. 5). Firstly, increased toxicity of either combination was excluded by crystal violet staining (Fig. 5A).

Significant TF reduction was achieved with both regimens (Fig. 5B–D, *p* < 0.001 vs. 5-FU monotherapy). As determined by immunofluorescence and flow cytometry, the SEQ combination even tended to reduce surface-bound TF more effectively than the SIM approach. Procoagulant activity was also reduced (Fig. 5E). Moreover, a significant anticoagulant effect of abemaciclib was observed after SEQ, but not after SIM treatment (vs. control). Again, the effects were independent of p27^kip1^-driven cell cycle arrest or senescence, as only minor changes were observed in either regimen (Supplementary Fig. 3).

To further investigate the specific effect of CDK4/6 inhibition on TF abundance and coagulation, the experiments were repeated with palbociclib. Again, SIM and SEQ combinations were tested (Fig. 5D,E). Interestingly, palbociclib had comparable effects on these two markers, but the effects were lower compared to abemaciclib. Therefore, we focused on abemaciclib alone for a more in-depth functional analysis of the underlying mechanisms of anticoagulation.

### The reduced procoagulant activity of abemaciclib is partially attributable to phosphatidylserine translocation on CRC cells

**Figure 6:**
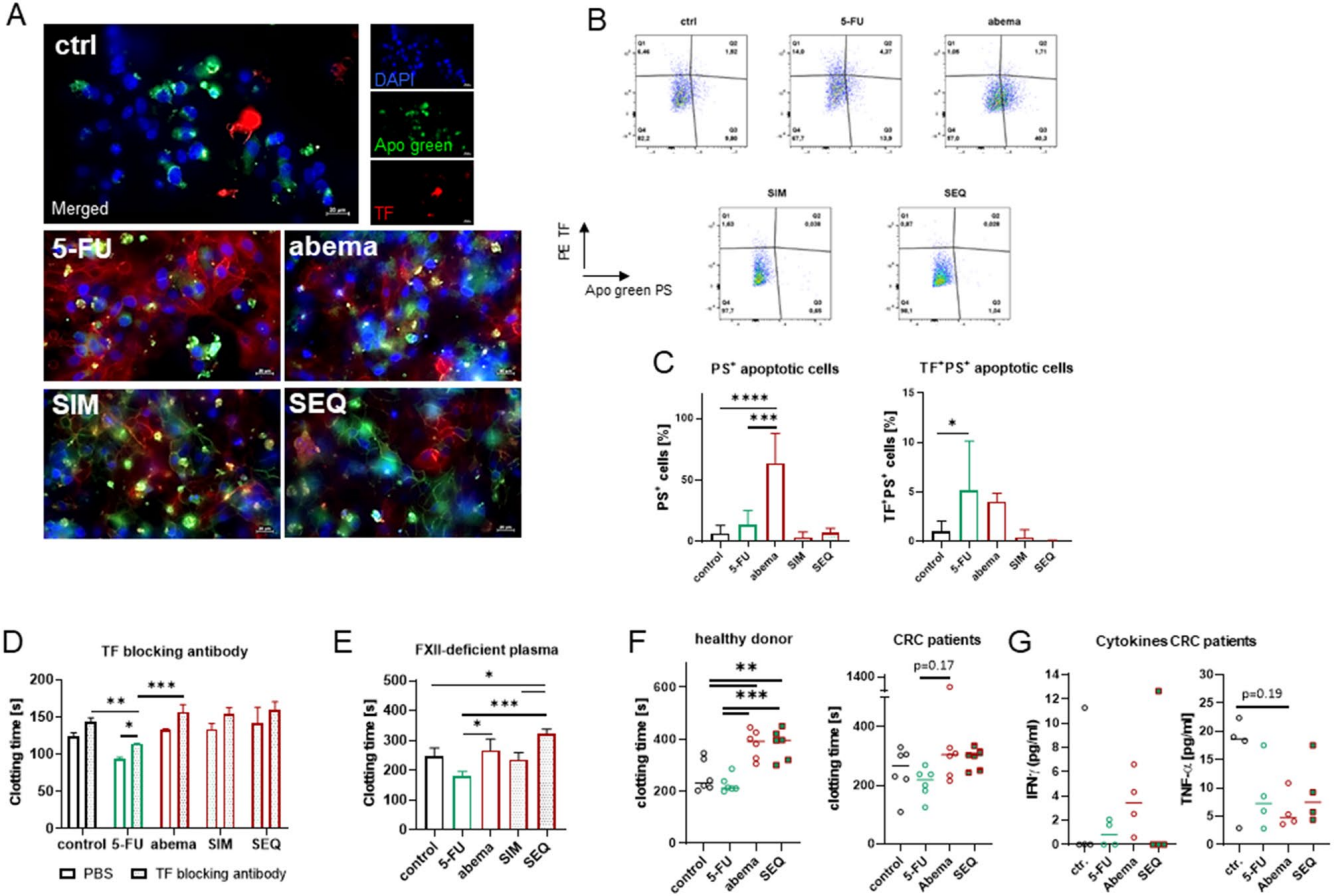
Phosphatidylserine-TF co-localization, TF blocking antibody, FXII-deficient plasma and PBMC coagulation. (**A**–**C**) Translocation of phosphatidylserine (PS) after treatment (5-FU 1 µM, abemaciclib 1 µM) was studied by (**A**) immunofluorescence and (**B**) flow cytometry. (**A**) Representative images are shown. Original magnification ×200. (**B**,**C**) Flow cytometry, n ≥ 3; mean + SD; one-way ANOVA (Tukey's multiple comparison test) ****p < 0.0001; ***p < 0.001; *p < 0.05 versus control. (**D**,**E**) Coagulation time using (**D**) a TF blocking antibody or (E) FXII-deficient *vs*. normal plasma, n ≥ 3; mean + SD; one-way ANOVA (Tukey's multiple comparison test). ***p < 0.001; **p < 0.01; *p < 0.05 versus control. (**F**) Coagulation time of PBMCs. N = 4–6 independent donors/group; median; one-way ANOVA (Tukey’s multiple comparison test). ***p < 0.001; **p < 0.01 versus control. (**G**) Cytokine secretion was quantified by ELISA. N = 4 independent donors/group, median, one-way ANOVA (Tukey's multiple comparison test).

The procoagulant phenotype of CRC cells is primarily dependent on TF and phosphatidylserine (PS) translocation to the outer membrane. In particular, PS is involved in the decoding of TF and acts as a driving force for the propagation of coagulation. Since TF up-regulation has been identified as the major procoagulant factor in the context of CRC, we examined translocated PS on the surface of tumor cells (Fig. 6A–C).

The number of PS-positive cells increased after treatment. This was particularly observed in cells exposed to abemaciclib (*p* < 0.0001 vs. control, *p* < 0.001 vs. 5-FU), while 5-FU and the combinations only slightly induced PS translocation. We then quantified the amount of double positive cells, i.e. the co-localization of TF and PS (Fig. 6A, C). Using immunofluorescence, double positive cells were primarily identified after 5-FU exposure, but not after abemaciclib mono- and combination treatment (Fig. 6A). Flow cytometric analysis principally confirmed these findings and abemaciclib significantly triggered PS translocation (Fig. 6B,C left). Looking at the double positive cell fraction, we only observed a significant increase when cells were exposed to 5-FU, while abemaciclib either alone or in combination with 5-FU abrogated this effect. This supports the assumption that PS is involved in TF decryption and that 5-FU triggers the procoagulant behavior in tumor cells. Notably, the amount of TF^+^PS^+^ cells was lowest in the SEQ combination, hence adding abemaciclib after 5-FU successfully counteracted TF-upregulation (Fig. 6B,C), while preserving its antitumoral potential (please see Fig. 5A).

Using a TF-blocking antibody, we formally confirmed the impact of TF on the procoagulant activity of CRC cells (Fig. 6D). The clotting time of HROC173 was significantly prolonged after blocking TF of 5-FU-treated cells. As anticipated, no significant changes were seen in samples treated with abemaciclib either alone or in combination (Fig. 6D). Then, we analyzed the procoagulant activity in FXII-deficient plasma (Fig. 6E). Here, the results were principally the same, meaning a significant shortening of cell-induced clotting time after treatment with 5-FU, and a prolongation after treatment with abemaciclib. Both SIM and SEQ schedules reduced the procoagulant activity of cells, reflected by significantly prolonged plasma coagulation times in FXII-deficient plasma.

As TF is produced also by immune cells, we tested whether treatment of PBMCs with 5-FU or abemaciclib might influence their procoagulant potential. Notably, treatment with 5-FU did not influence coagulation time, however, abemaciclib and the SEQ regimen significantly prolonged coagulation time of immune cells from healthy donors (Fig. 6F left). Then, PBMCs from CRC patients were tested. In contrast to healthy donors, the samples from the patients (with advanced CRC disease) were collected after surgery and before the start of adjuvant treatment. A trend toward prolonged plasma coagulation was observed when PBMCs were exposed to abemaciclib (Fig. 6F, right), however the effect was not significant. Additional cytokine profiling identified slightly elevated levels of IFNγ and reduced levels of TNFα after abemaciclib, thus supporting its influence on immune and coagulation response (Fig. 6G).

### Secretion of procoagulant microvesicles upon treatment

We then purified microvesicles (MVs) from treated and control cells (Supplementary Fig. 3C,E). MVs are cell-derived membranous structures that have been used as biomarkers to assess the risk of developing venous thromboembolism (VTE)^25–27^. Data in CRC patients remain controversial, but most studies show no direct association between MV-TF activity and VTE.

Here, we quantified the number of MVs using Nanosight to determine whether mono- or combined therapy affected the number of MVs secreted. Only in the SIM setting were significantly more MVs detected compared to the control (Supplementary Fig. 3C). Notably, the size of MVs did not differ significantly between treatment schedules (Supplementary Fig. 3D). Subsequently, the procoagulant activity was determined and thus, TF was indirectly analyzed as a prothrombotic factor. MVs induced coagulation when added to recalcified plasma, but no significant differences were observed between groups (Supplementary Fig. 3E). Thus, although the number of MVs was significantly increased with SIM therapy, their procoagulant activity did not change significantly. Therefore, we conclude that the role of (procoagulant) MVs is of minor importance, at least in this model.

## Discussion

In this study, we report an upregulation of the procoagulant TF on CRC cells after a single short-term treatment at subtherapeutic doses, which was counterregulated by selective, but not pan-CDK inhibition.

Systemic hypercoagulability in cancer patients and TF overexpression by cancer cells are closely associated with tumor progression and poor patient prognosis^1^. Oncogenic events trigger TF overexpression and coagulopathy, which in turn promotes proliferation and metastasis through the activation of the intracellular protease-activated receptor-2 signaling pathway ultimately allowing disseminating cells to escape immune surveillance^13^. Chemotherapy, such as 5-FU, is a well-established risk factor for VTEs, which is classified as grade 3/4 toxicity in CRC patients receiving chemotherapy in the outpatient setting^5^. Here, we add further credence to the procoagulant activity of 5-FU. Upregulation of TF, together with reduced plasma coagulation time, occurred after short-term treatment with non-toxic doses. Functionally, this is due to an imbalance between coagulation activators (= TF) and inhibitors (= proteins C/S)^5^. This direct association between chemotherapy and VTE certainly increases with the number of chemotherapy cycles. GEM, another drug commonly used in the clinical setting, had comparable procoagulant effects on CRC cells. However, the effect on TF expression was less than that of 5-FU with no sustained effect. In both cases, TF^+^ cells showed signs of EMT, such as a high expression of vimentin, fibronectin, and mucin accompanied by slightly reduced levels of E-Cadherin. This so-called “cadherin switch” during EMT represents a cell survival strategy^28^. It enhances the migratory and invasive properties of cancer cells and triggers chemoresistance via the context-dependent mesenchymal status and the dysregulation of certain transcription factors^29,30^. Concurrently, drug-resistant cells upregulate TF and exhibit a hypercoagulable state. This switch from a proliferative to an invasive phenotype explains the complications seen in patients receiving chemotherapy. A previous study already described EMT in rectal cancer samples, driven by senescent cancer cells after neoadjuvant chemoradiotherapy^31^. In cervical cancer, the TF-PAR-2 pathway mediates cisplatin resistance through an epidermal growth factor receptor (EGFR)- and COX2-dependent mechanism^32^. In vitro analysis revealed TF up-regulation by EGF treatment of cervical cancer cells, whereas treatment with cetuximab decreased TF protein levels. Similarly, in glioblastoma, EGFR/EGFRvIII overexpression increases TF expression^33,34^. The specific JNK inhibitor SP600125 counteracts this upregulation^33^. In CRC, TF is under the control of two major transforming events that drive disease progression (*KRAS* and *TP53* mutation) in a MEK/mitogen-activated protein kinase and PI3K-dependent manner^10^. Here, despite the fact that we analyzed two different cell lines, we indeed observed that chemotherapy-induced TF upregulation was higher in the *KRAS*^mut^ cells than in the *KRAS*^wt^ cells, whose TF expression was only altered by gemcitabine, but not by 5-FU. Since 5-FU and GEM have different effects on cell signaling pathways and mechanisms, it is tempting to speculate that the observed differences between these two cell lines after 5-FU exposure are—at least in part—related to the *KRAS* status, whereas the effect of GEM could be due to different, possibly even post-transcriptional (protein stabilizing) events, which we did not investigate further here. This supports the notion that the crosstalk between TF/FVIIa and EGFR signaling pathways in CRC is dependent on *KRAS* mutation^35^. Indeed, another recent publication confirmed our hypothesis of a *KRAS* mutant-driven hypercoagulable state in colorectal cancer cell lines (vs. their Kras^wt^ counterpart)^36^. Given that approximately half of all CRC cases harbor a *KRAS* mutation (~ 45%^37^), this may be of particular clinical relevance. No association was found for *TP53*, as both cell lines harbored at least one pathogenic *TP53* mutation.

Pharmacological antagonists of some of these transforming genes (e.g., EGFR inhibitors) could reduce TF expression, both locally and systemically, and these targeted agents are considered as potential indirect and cancer-specific anticoagulants, in addition to their direct antitumor effects^38^. In this study, we tested CDKIs as potential TF counter-regulators. The two CDK4/6 inhibitors abemaciclib and palbociclib abrogated 5-FU-induced TF up-regulation and associated procoagulant properties. As shown here for abemaciclib, this effect was independent of senescence and cell cycle arrest, contrasting with previous reports showing senescence-associated TF abrogation^23^. It has been described that p27^Kip1^ reduces TF expression in endothelial cells in vitro by inhibiting the TF promoter activity^22^. P27^Kip1^ is physiologically expressed in the G1 phase and induces a cell cycle arrest^39,40^. TF is mainly found on highly proliferating cells. Hence, an inverse expression pattern of p27^Kip1^ and TF might be expected. Here, such a correlation was only observed for abemaciclib, which was characterized by a significant prevention of TF-upregulation and a slight upregulation of p27^Kip1^. However, we could not confirm this inverse expression pattern for the other compounds tested. Due to their mode of action, CDKIs activate p27^Kip1^ to induce cell cycle arrest^41^, which complicates data interpretation. The notion that p27^Kip1^ was higher with dinaciclib and abemaciclib suggests a minor direct impact of p27^kip1^ on TF downregulation.

Other interesting findings were reduced TF levels and prolonged coagulation times. This was mainly observed with sequential and, to a lesser extent, simultaneous treatment with 5-FU and abemaciclib or palbociclib. This finding identified CDK4/6 inhibitors as promising add-on agents for the treatment of CRC to reduce the risk of developing TF-mediated thromboembolic events. The exact underlying mechanism of CDK4/6-blocking mediated TF downregulation is currently unknown. The fact that palbociclib, unlike abemaciclib, specifically inhibits CDK4/6, but has comparable effects on the CRC coagulation phenotype in our present study, suggests a role for these two cell cycle regulators in TF suppression. Hence, TF is the key driver of the tumor cells’ hypercoagulable phenotype. Using a TF blocking antibody, the procoagulant activity of 5-FU treated cells was significantly impaired. In contrast, no significant changes were observed in cells treated with abemaciclib alone or in combination, consistent with reduced hypercoagulability. Moreover, in FXII-deficient plasma, the effects were essentially the same as in normal plasma, supporting the notion that TF plays an important role in activating coagulation. TF and translocated PS from apoptotic cells increase the risk of developing VTEs^25^. Here, we found higher levels of cells with co-localized TF and PS only after SIM treatment, which is consistent with the above findings of adverse effects after combined 5-FU/abemaciclib treatment. When this approach was extended to circulating immune cells, the same coagulation results were observed. PBMCs from healthy donors and CRC patients showed prolonged coagulation times after abemaciclib monotherapy and the SEQ combination. Functionally, this was due to reduced levels of TNFα, which is associated with acquired hypercoagulability^42^, and may be used as a biomarker to estimate VTE risk.

VTEs can be localized or disseminated. In this study, we identified local rather than systemic effects of short-term 5-FU treatment. The amount of secreted TF was not elevated after a single 5-FU application, but was again lowest after abemaciclib mono- and SEQ combination. Cancer cells often secrete large amounts of MVs. In particular, TF-bearing MVs can be highly elevated in patients undergoing chemotherapy and contribute to hypercoagulability^8,43^. Mechanistically, this is due to increased binding and induction of coagulation factors and platelet aggregation by procoagulant MVs. TF blockade prevented physical interaction of platelets with CRC cells in one study, while others report a minor role of TF blockade in suppressing coagulation^7,26^. It is tempting to speculate that both the extrinsic (= TF) and intrinsic (= FXII) coagulation cascades are involved in initiating coagulation in CRC patients. Here, we observed slight differences in number, size, and clotting time of MVs from CRC cells. A previous study reported enhanced chemotherapy-induced procoagulant activity of human lymphoblastic leukemia cells via extracellular vesicles^44^. Cells were treated with high doses of vincristine for 4 or 24 h. Comparable effects can be expected after high-dose treatment and shorter follow-up.

While prophylactic anticoagulation appears to have a positive impact on patient outcomes, there is still no consensus on the agent and timing to prevent an increase in major bleeding. Recently, the FDA approved Tisotumab vedotin, an antibody–drug conjugate designed to target TF-expressing cancers, without affecting blood coagulation^45,46^.

To the best of our knowledge, this is the first study to describe TF upregulation after low-dose chemotherapy in CRC cells. The chemotherapy-induced TF^+^ cells had increased procoagulant activity, which could be counteracted by abemaciclib—a promising adjuvant therapy in CRC to reduce the risk of developing TF-mediated thromboembolic events.

## Methods

### Cell culture

Low-passage human CRC cell lines HROC173 and HROC257 T0 M1 were obtained from Cell line services (Eppelheim, Germany). The HaCat cell line was originally obtained from the German collection of cell cultures (DSMZ; Braunschweig, Germany) and routinely cultured. Cells were maintained in DMEM/HamsF12 supplemented with 10% fetal calf serum (FCS), glutamine (3 mmol/L) and antibiotics (medium and supplements were purchased from PAA, Cölbe, Germany). Mycoplasma exclusion was done regularly (every 3–4 passages) by DAPI staining.

### Drug doses & treatment duration

The following drugs were used: 5-FU (1.0 µM, + dose-finding with 0.1 and 0.5 µM, respectively), gemcitabine (GEM, 0.15 µM, + dose-finding with 0.02 and 0.075 µM, respectively), and the CDKIs dinaciclib (stock: 10 mM, working concentration: 0.01 µM), abemaciclib (stock: 10 mM, working concentration: 1 µM), THZ-1 (stock: 10 mM, working concentration: 0.03 µM), and palbociclib (stock: 10 mM, working concentration: 1 µM). Cytostatics 5-FU and GEM were obtained from the central pharmacy at the UMR. CDKIs were obtained from MedChemExpress (Cologne, Germany). Cells were either treated for 48 or 72 h. In some cases, drug combinations were applied. These were either given simultaneously (SIM) or sequentially (SEQ). The concentrations were the same as in the monotherapy. The SIM approach was done for 48 h and the SEQ approach for 96 h.

### Crystal violet staining and wound healing assay

Viability after drug treatment was analyzed by crystal violet staining as described before^47^. Tumor cell migration at baseline and after chemotherapy-induced TF-upregulation was investigated as described before^48^.

### Flow cytometry

CRC cell lines were treated with 5-FU (1.0 µM) or gemcitabine (GEM) (0.15 µM) 48 or 72 h. 0.5 × 10^6^ cells were stained with PE mouse anti-human CD142 antibody (TF, 1 µg, BioLegend clone: NY2, Koblenz, Germany) or isotype control (BioLegend, PE Mouse IgG1, κ Isotype Ctrl (FC)) according to the manufacturer’s instructions (20 min, RT). DAPI (3 µM, BioLegend) was used for dead cell exclusion. Cells were measured on a BD FACSAriaIIU (25 mW 405 nm-laser, 30 mW 561 nm-laser, 20 mW 488nm-laser, 11mW 633nm-laser) using the 450/40 detector for DAPI or the 582/15 detector for PE, respectively. After doublet discrimination, DAPI^−^ and CD142^+^ cells were recorded. In some experiments, APC-Vio 770-labeled anti-E-Cadherin (Miltenyi Biotec, clone 67A4) and PE/Cyanine7-labeled anti-N-Cadherin (BioLegend, clone 8C11) antibodies were used at 0.2µM.

### Cell sorting approach

PE anti-CD142 labeled CRC cells, either treated with 5-FU (1.0 µM) or GEM (0.15 µM), were stained with DAPI. Based on the mean fluorescence intensity (MFI), the top 20% CD142-positive cells were bulk sorted on a BD FACSAria™IIIu. Sorting was performed using a 100 µm nozzle (efficiency of > 90%) Data acquisition was done with BD FACSDiva™-software v9.4.

### Clotting assay

Cells (1 × 10^5^) were harvested, washed and incubated with 100 µL plasma (1 min, 37 °C) in a coagulometer, supplemented with 25 mM CaCl_2_ to activate clotting. In some experiments, cells were pre-incubated with a TF blocking antibody. Additional clotting experiments were done with peripheral blood mononuclear cells (PBMC) from healthy donors or CRC patients after surgery, prior to adjuvant therapy. Patient consent was obtained in all cases. All procedures were approved by the Ethics Committee of the *Rostock University Medical Center* (ref. no. II HV A 2017-0191) following generally accepted guidelines for the use of human material. PBMCs (1 × 10^6^ cells/condition) were incubated with 5-FU, abemaciclib (1 µM each), or left untreated. For the SEQ combination approach, cells were treated with 5-FU for 8 h, washed and exposed to abemaciclib. Clotting was done as described.

### RNA Isolation, cDNA synthesis, and quantitative real-time PCR

Total RNA was isolated using the RNeasy Kit (Qiagen). Target cDNA levels were analyzed by quantitative real-time PCR using predesigned TaqMan gene expression assays either labeled with 6-FAM-3′ BHQ-1 or 5′ HEX-3′ BHQ-1: F3, CTGF, TFPI, VIM, MUC-1, FN1, F9, and PLAU. GAPDH was used as housekeeping gene. Reaction was performed 12.5 ng cDNA as follows: 95 °C, 10 min, 40 cycles: 15 s at 95 °C, and 1 min at 60 °C. All reactions were run in triplicates. The mRNA levels of target genes were normalized to GAPDH. Expression level of each sample was considered by calculating 2^−ΔΔCT^ (ΔCT_target_−ΔCT_Calibrator_). Untreated cells were used as calibrator.

### Immunofluorescence staining

Cells were grown in ibidi chamber slides (Gräfelfing, Germany), fixed, permeabilized, blocked with 10% serum and incubated overnight with primary anti-human antibodies: rabbit Claudin-7 (1:100, Thermo Fisher Scientific, Dreieich, Germany), rabbit E-Cadherin (1:100, Santa Cruz Biotechnology, Heidelberg, Germany), followed by a secondary AF488® goat anti-rabbit antibody (1:1000, 1 h, Molecular Probes, Darmstadt, Germany). Directly labeled antibodies were used: PE mouse CD142 antibody (1:50), AF488® mouse CD326 (EpCAM) Antibody (1:100, BioLegend), AF594® N-Cadherin (1:100, Wiesbaden, Germany), AF488® anti-p27/Kip1 Antibody (1:50, Novus Biologicals), AF594® mouse p53 Antibody (1:250, BioLegend), AF488® rabbit p21 Waf1/Cip1 Antibody (1:300, Cell Signaling Technology, Frankfurt am Main, Germany), AF546® mouse p16 Antibody (1:50, Santa Cruz Biotechnology). Nuclei were counterstained with DAPI. Cells were analyzed on a Zeiss Axio Observer 7 Microscope using a Axiocam 702 mono camera and the ZEN pro software.

### Microvesicle purification, MV quantification by Nanoparticle Tracking Analysis (NTA)

Purification of microvesicles (MVs) was done as described^49–52^. Cell culture supernatant was characterized using a NanoSight® LM 10 instrument (Malvern Instruments Ltd., Worcestershire, UK) as described^51^. MV concentrations were calculated by counting the particles in the known volume of observed field. Medium was used as a control.

### Phosphatidylserine exposure and TF abundance

Tumor cells were stained with Apotracker™ Green (0.2 µM, Biolegend) for detecting PS-translocation and with the PE mouse anti-human CD142 antibody to quantify TF as mentioned above. Nuclei were visualized with DAPI. Flow cytometric measurements were done on a Cytek™ Aurora (VBR-lasers). Data were quantified with FlowJo 10.6.1.

### Replicates

All experiments were at least repeated three times with one–three technical replicates. Image J quantification of immunofluorescence images was done from three individual images per biological replicate with a total of three–four biological replicates.

### Statistical analysis

All values are reported as mean ± SD. After proving the assumption of normality (Shapiro–Wilk test), differences between controls and treated cells were determined by applying one way ANOVA on ranks (Bonferroni`s Multiple Comparison Test or Tukey’s multiple comparisons test). The criterion for significance was taken to be *p* < 0.05 as follows: *p < 0.05, **p < 0.01, ***p < 0.001, ****p < 0.0001 and #*p* < 0.05, ##*p* < 0.01, ####*p* < 0.0001 versus untreated cells.

### Ethics statement

Approval of the research protocol by an Institutional Reviewer Board: Ethics Committee of the Rostock University Medical Center, University of Rostock (ref.-no: II HV A 2017-0191).

### Informed consent

Obtained from all participants.

### Supplementary Information

Supplementary Information 1.

Supplementary Information 2.

Supplementary Information 3.

Supplementary Information 4.

## Data Availability

The datasets used and/or analyzed during the current study are available from the corresponding author on reasonable request.
