# Supplementary figures and images for: Selective but not pan-CDK inhibition abrogates 5-FU-driven tissue factor upregulation in colon cancer
Source: Sci Rep. 2024 May 8;14:10582. doi: 10.1038/s41598-024-61076-5 (PMC11078971; doi:10.1038/s41598-024-61076-5)

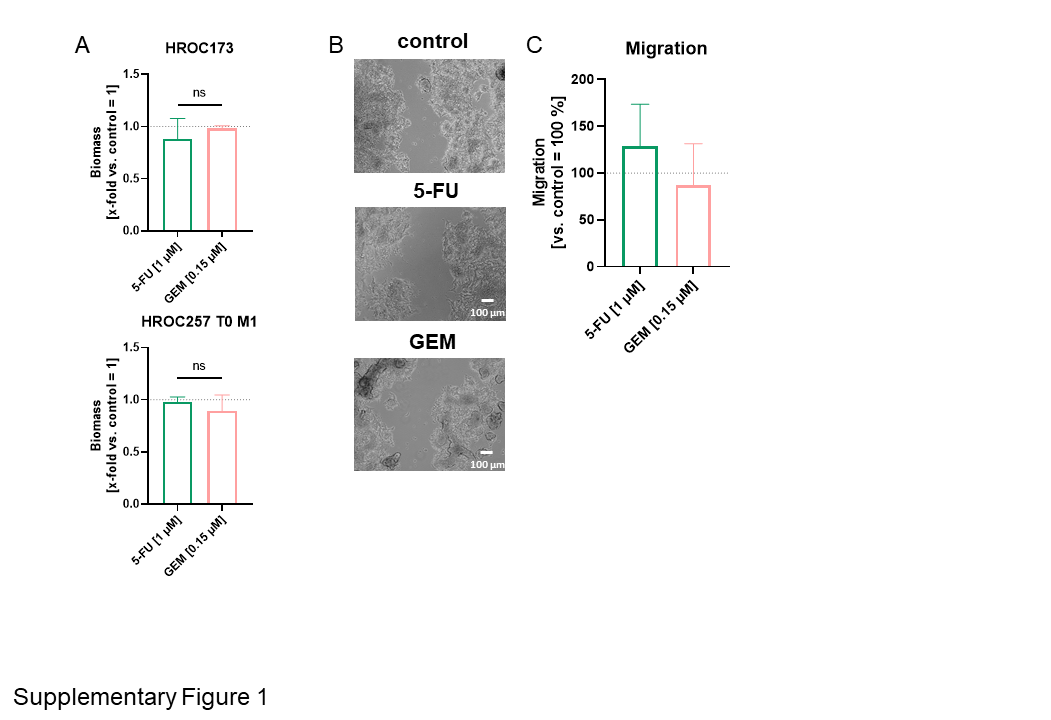

Supplement: Supplementary file 1 — Supplementary Information 1. [file 41598_2024_61076_MOESM1_ESM.tif]

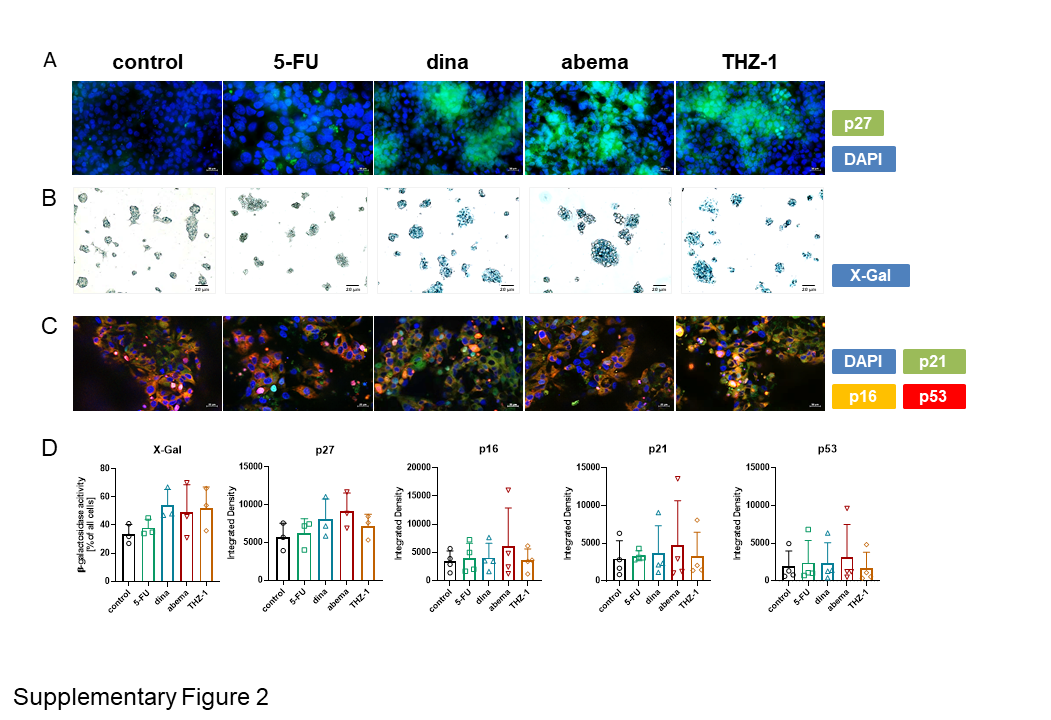

Supplement: Supplementary file 2 — Supplementary Information 2. [file 41598_2024_61076_MOESM2_ESM.tif]

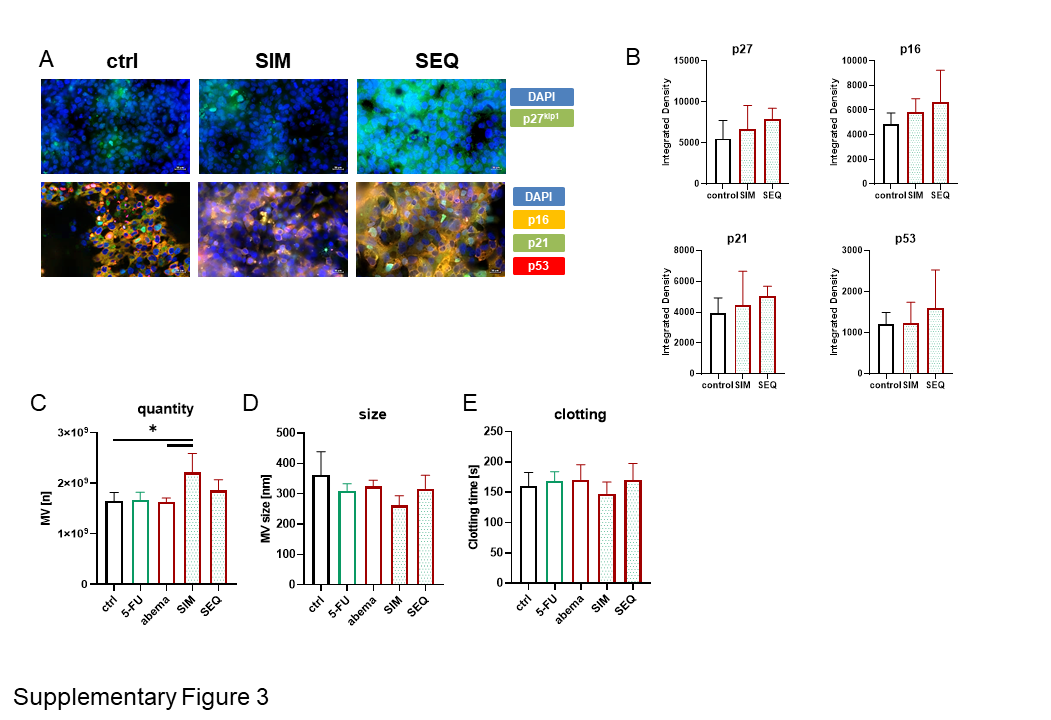

Supplement: Supplementary file 3 — Supplementary Information 3. [file 41598_2024_61076_MOESM3_ESM.tif]
