# Supplementary material for: Selective but not pan-CDK inhibition abrogates 5-FU-driven tissue factor upregulation in colon cancer
Source: Sci Rep. 2024 May 8;14:10582. doi: 10.1038/s41598-024-61076-5 (PMC11078971; doi:10.1038/s41598-024-61076-5)
Supplement: Supplementary file 4 — Supplementary Information 4. [file 41598_2024_61076_MOESM4_ESM.docx]

**List of Supporting Information:**

**Supplementary Figure 1: Cytotoxic effects of the used drugs were excluded by two different methods.** (A) CRC cells (HROC173, HROC257 T0 M1) were treated with 5-FU (1 µM) or GEM (0.15 µM) for 48h. Control cells were left untreated. Viability of CRC tumor cell lines was analyzed by crystal violet staining. All data were calculated in relation to the control, which was set to be =1, n= 3; mean + SD. (B, C) A wound healing was done with HROC173 cells treated with 5-FU (1 µM), GEM (0.15 µM), or left untreated. Data acquisition was done at day 2 (= 48 h). (B) Representative images are shown. Original magnification 200x. (C) Data quantification as determined by measuring the length at the longest point/well. All data were calculated in relation to the control, which was set to be =100%, n= 3; mean + SD.

**Supplementary Figure 2: Impact of 5-FU and CDKI treatment on senescence and cell cycle arrest.** Tumor cells were either left untreated or exposed to 5-FU (1 µM) and CDKIs (dinaciclib 0.01 µM, abemaciclib 1 µM, and THZ-1 0.03 µM) for 48 hours. (A) Immunofluorescence. (B, C) Senescence was studied by (B) beta-galactosidase staining and (B) immunofluorescence (p16, p21, p53). (A-C) Representative images of tumor cells. (D) Quantification, n≥ 3. mean + SD.

**Supplementary Figure 3:** Impact of SIM and SEQ treatment on senescence, cell cycle arrest, and microvesicles. (A, B) Immunofluorescence was done to study cell cycle arrest (p27) and senescence induction (p16, p21, p53) upon SIM and SEQ 5-FU (1 µM) and abemaciclib (1 µM) treatment. (A) Representative images. (B) Quantification, n≥ 3; mean + SD. (C – E) MVs were isolated from cell culture supernatants as described in material & methods. (C) Supernatants of cells were analyzed by Nanosight and the number of EVs was calculated. n≥ 3; mean + SD; one-way ANOVA (Tukey's multiple comparison test). *p<0.05 *vs*. control. (D) Size of MVs was determined with NTA, n≥ 3;mean + SD. (E) Purified MVs were added to recalcified plasma and the time until clot formation measured in a coagulometer, n≥ 3, mean + SD.
